# Diaphragmatic downward excursion as a novel metric for assessing Valsalva maneuver efficacy in patent foramen ovale detection by contrast transthoracic echocardiography

**DOI:** 10.3389/fcvm.2025.1616241

**Published:** 2025-12-19

**Authors:** Yun Li, Anni Chen, Jianbo Zhu, Lei Zhu, Turgunov Boburjon, Zhenzhen Jiang, Xiatian Liu

**Affiliations:** 1 Department of Medicine, University of Shaoxing, Shaoxing, China; 2 Department of Ultrasound, Shaoxing People’s Hospital, Shaoxing, China; 3 Department of Ultrasound, The First Affiliated Hospital of Shaoxing University, Shaoxing, China; 4 Republican Specialized Center of Surgery Named after Academician V. Vakhidov, Tashkent, Uzbekistan

**Keywords:** patent foramen ovale, diaphragm, Valsalva maneuver, right-to-left shunt, transthoracic echocardiography, transesophageal echocardiography, saline contrastechocardiography

## Abstract

**Objective:**

Contrast transthoracic echocardiography (c-TTE) is widely used for the diagnosis of patent foramen ovale (PFO), where the Valsalva maneuver (VM) serves as the standard provocative maneuver to optimize detection. This study aimed to evaluate diaphragmatic downward excursion (DDE) as a novel c-TTE–based parameter for objectively quantifying VM efficacy, thereby establishing a standardized assessment metric.

**Methods:**

We studied 145 patients with high clinical suspicion of PFO-related conditions. All participants underwent both c-TTE and contrast transesophageal echocardiography (c-TEE) examinations. Based on intraoral expiratory pressure exceeding 40 mmHg under c-TTE, patients were divided into adequate Valsalva maneuver (AVM) group (*n* = 90) and non-adequate Valsalva maneuver (non-AVM) group (*n* = 55). We compared the two groups in terms of DDE at the roof of the right atrium (DDE-RRA) and intracardiac hemodynamic parameters.

**Results:**

DDE-RRA was significantly lower in the AVM group than in the non-AVM group (7.3 mm vs. 3.1 mm, *P* < 0.001). ROC analysis identified 5 mm as the optimal cutoff value for evaluating VM efficacy, with a sensitivity of 77.8%, specificity of 92.7%, and an AUC of 0.90. The kappa test showed good agreement between DDE-RRA and insufflation manometry (kappa = 0.63, *P* < 0.001). Furthermore, the DeLong test demonstrated that the AUC of DDE-RRA was significantly greater than that of all assessed intracardiac hemodynamic parameters, including mitral and tricuspid peak E and A-wave velocities, as well as mitral and tricuspid velocity time integrals (all *P* < 0.05).

**Conclusion:**

DDE provides a simple and objective method for assessing VM efficacy under c-TTE, showing superior diagnostic performance compared with conventional intracardiac parameters. As this represents an initial attempt, further studies incorporating invasive validation are needed to confirm its clinical value.

## 1 Introduction

Patent foramen ovale (PFO) is a potential gap of incomplete primary and secondary septal fusion after 3 years of birth, with an incidence of around 25% in the general population (1). PFO may be associated with multiple diseases and symptoms, including cryptogenic stroke, transient ischemic attack, migraine, and hypoxemia (2–5). Hence, accurate detection of PFO is of enormous clinical significance. While contrast transesophageal echocardiography (c-TEE) is widely regarded as the most accurate diagnostic method for detecting PFO, its status as the “gold standard” remains contested. c-TEE is semi-invasive, patients with oral-esophageal probes tend to be unable to complete adequate provocative maneuvers due to discomfort. Compared to c-TEE, patients can perform better provocative maneuvers under contrast transthoracic echocardiography (c-TTE). It has been shown that although c-TTE is less accurate than c-TEE in detecting PFO, c-TTE is more sensitive with adequate provocative maneuvers (6). Therefore, c-TTE is commonly used in the clinical routine as a highly efficient technique for PFO screening.

Performing effective provocative maneuvers is crucial for PFO detection. Valsalva maneuver (VM) is the most widely utilized technique to diagnose PFO. VM is used during examinations to transiently increase right atrial pressure, displacing the primary septum of the fossa ovalis toward the left atrium. This increases the separation between the primary and secondary septa, facilitating the opening or enlargement of a PFO. As a result, microbubbles indicating a right-to-left shunt (RLS) can be detected immediately after VM release. However, inadequate VM may fail to raise right atrial pressure sufficiently, leading to false-negative results and underestimating the severity of RLS. Traditional VM requires deep inspiration, mouth and nose closure, and forced expiration. The modified VM omits deep inspiration and involves only mouth and nose closure with rapid forced expiration. This modification improves image stability, allowing clearer visualization of the microbubbles passing through the PFO. The execution of an effective VM is characterized by indicators such as jugular venous distension, face flushing, heightened abdominal muscle tone etc. Additionally, blood pressure and heart rate also change dynamically during different stages of VM. However, these symptoms are difficult to quantify and qualify. Currently, the efficacy of VM is primarily evaluated through active insufflation manometry under c-TTE. VM is deemed effective if the patient achieves an insufflation pressure of 40 mmHg and maintains this level for a minimum of 10 s. However, this method requires active and forceful insufflation by the patient and external devices such as pressure gauges and disposable blowing pipelines that are not convenient enough. Hence, we attempted to find an objective and accurate indicator for assessing the efficacy of VM, without the need for additional devices. VM elevates thoracic pressure, resulting in diaphragmatic downward excursion (DDE). During our clinical practice, we found that the diaphragm located at the roof of the right atrium could be clearly visualized in the apical four-chamber view using transthoracic echocardiography (TTE), and its position would significantly synchronously move downward with VM. We propose that there may be a correlation between the DDE and the efficacy of VM. Simultaneously, VM can cause hemodynamic changes through a series of respiratory state changes. Our study recorded changes in relevant parameters of VM and assessed the diagnostic value of these parameters for the efficacy of VM.

## 2 Materials and methods

### 2.1 Patient population

**Figure 1:**
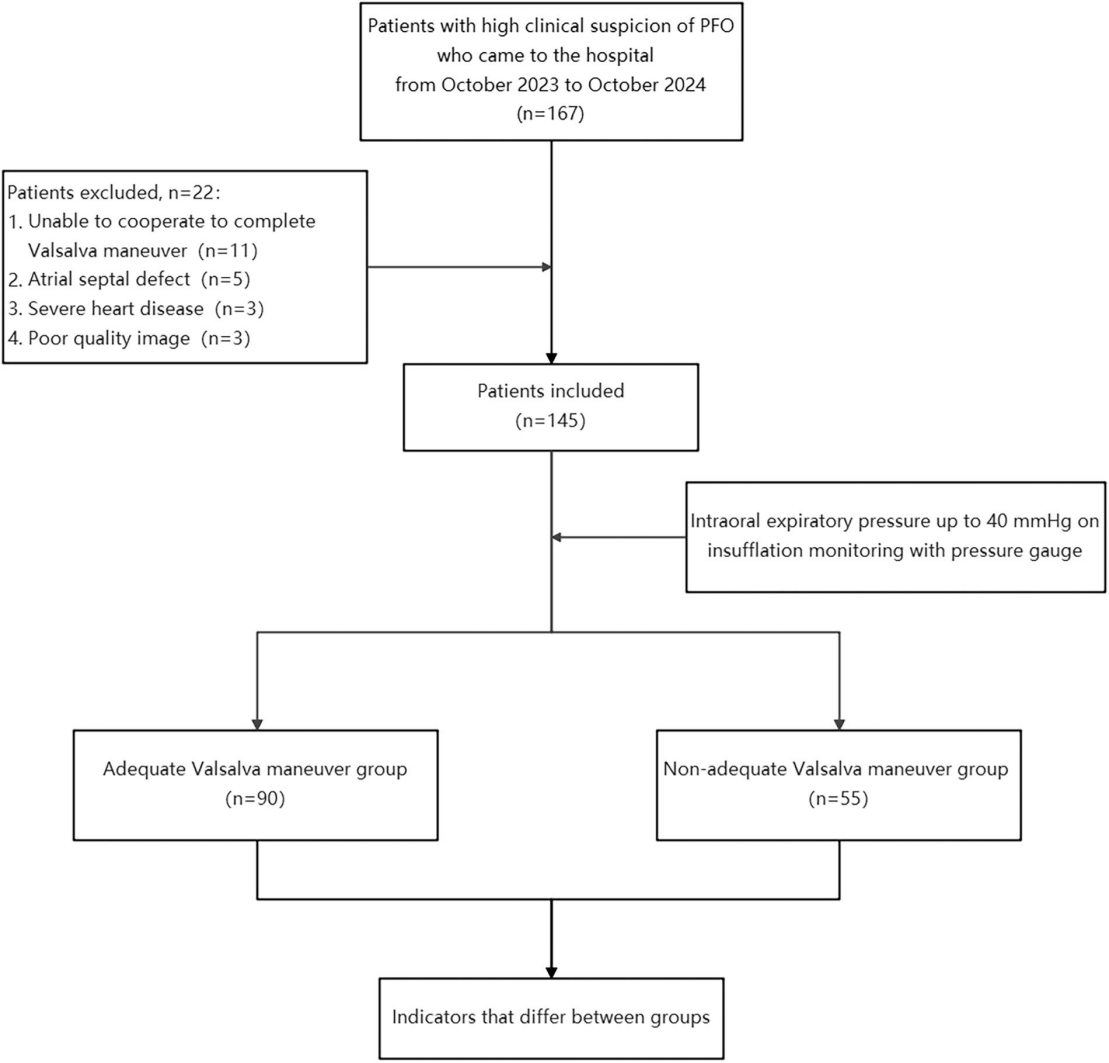
Flowchart. PFO, patent foramen ovale.

Our study prospectively investigated patients with a high clinical suspicion of PFO-related diseases between October 2023 and October 2024 at our hospital. Patients with atrial septal defect, severe heart disease, inability to cooperate with VM, poor image quality, or difficulty in measurement were excluded. Ultimately, 145 patients were included in the study. All included patients subsequently underwent further c-TEE examinations to confirm the presence of PFO. Based on intraoral expiratory pressure measurements under c-TTE, patients were divided into two groups: 90 patients in the adequate Valsalva maneuver (AVM) group and 55 patients in the non-adequate Valsalva maneuver (non-AVM) group (Figure 1). Informed consent was obtained from all participants. The study was officially approved by the Ethics Committee of Shaoxing People's Hospital.

### 2.2 Echocardiography

All patients underwent c-TTE and c-TEE examinations. The examination instrument was Philips EPIQ 7C (Philips Healthcare, Andover, Massachusetts, USA) equipped with an X5-1 probe at 1–5 MHz and an X8-2t probe at 3–8 MHz. The agitated saline mixture consisted of 1 mL of air, 1 mL of blood, and 8 mL of saline. Two 10 mL syringes were connected and agitated at a rate of 80 oscillations per minute before being swiftly administered through the antecubital vein.

Prior to the examination, patients were trained to perform appropriate VM. They were instructed to forcefully exhale against a closed glottis for more than 10 s without inhaling. Upon receiving instructions from the sonographer, the participants were instructed to release their exhalation rapidly. The training process may repeat 2–3 times to ensure the accuracy of the following c-TEE examination. The effectiveness of VM was assessed using insufflation manometry and DDE separately.

Patients under c-TEE were given local anesthesia in the oropharynx using 2% lidocaine gel. The criterion used to diagnose PFO under c-TEE was direct observation of microbubbles emerging from the foramen ovale. Each c-TTE was performed by two cardiac sonographers with more than 10 years of experience in TTE, each c-TEE was performed by two cardiologists with more than 5 years of experience specialized in echocardiography, and all diagnoses were performed by two cardiologists. When cardiologists disagreed about the diagnosis, we eventually invited other senior cardiologists who discussed it comprehensively and made the decision.

To evaluate inter-observer reproducibility, DDE-RRA was independently measured by two observers, each blinded to the other's results. Agreement was assessed using the intraclass correlation coefficient (ICC, two-way random-effects model, absolute agreement). The ICC was 0.912, indicating excellent reproducibility. In our experience, the acquisition of DDE-RRA is straightforward for trained echocardiographers, as it relies on standard apical four-chamber views without requiring additional imaging windows or patient repositioning. Therefore, minimal additional training is required, and the high ICC further supports the ease of adoption and clinical applicability of this method. Future multicenter studies incorporating structured training protocols could provide further validation of its reproducibility and generalizability across diverse clinical settings.

### 2.3 Parameter measurement and definition

**Figure 2:**
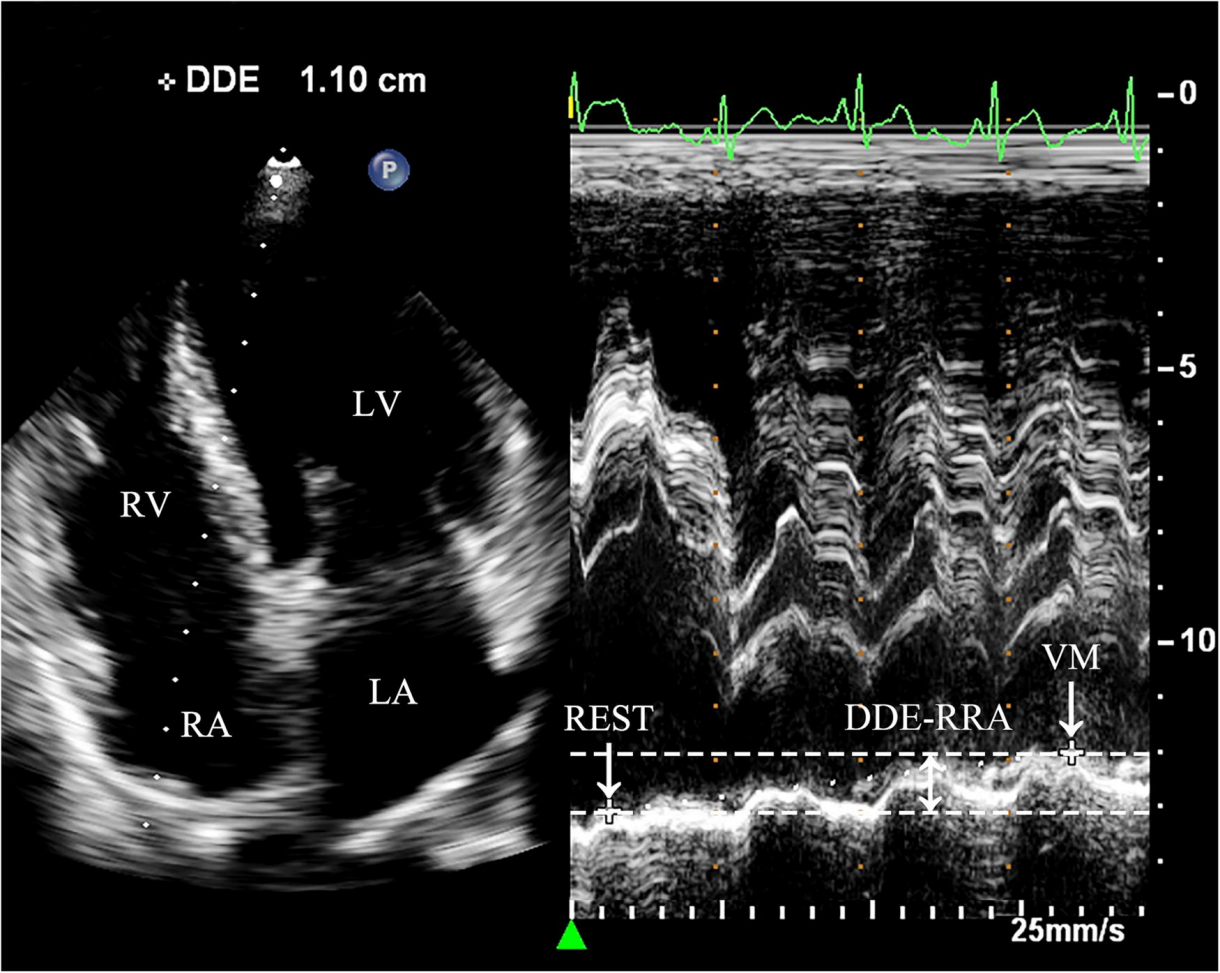
Measurement of DDE-RRA. Dramatic diaphragmatic downward excursion is observed. In the apical four-chamber view, the sampling line is placed across the top of the right atrium to obtain the M-mode motion curve of the diaphragm. REST refers to the moment right before the subject initiates the Valsalva maneuver, when they are in a baseline or resting state. VM refers to the point when the subject is holding their breath for 10 s during the Valsalva maneuver, just before releasing the breath. DDE-RRA (double-headed arrow) is the measured value representing the difference in the diaphragm's position between two distinct time points. REST, at rest; VM, Valsalva maneuver; LA, left atrium; LV, left ventricle; RA, right atrium; RV, right ventricle; DDE-RRA, diaphragmatic downward excursion at the roof of the right atrium.

**Figure 3:**
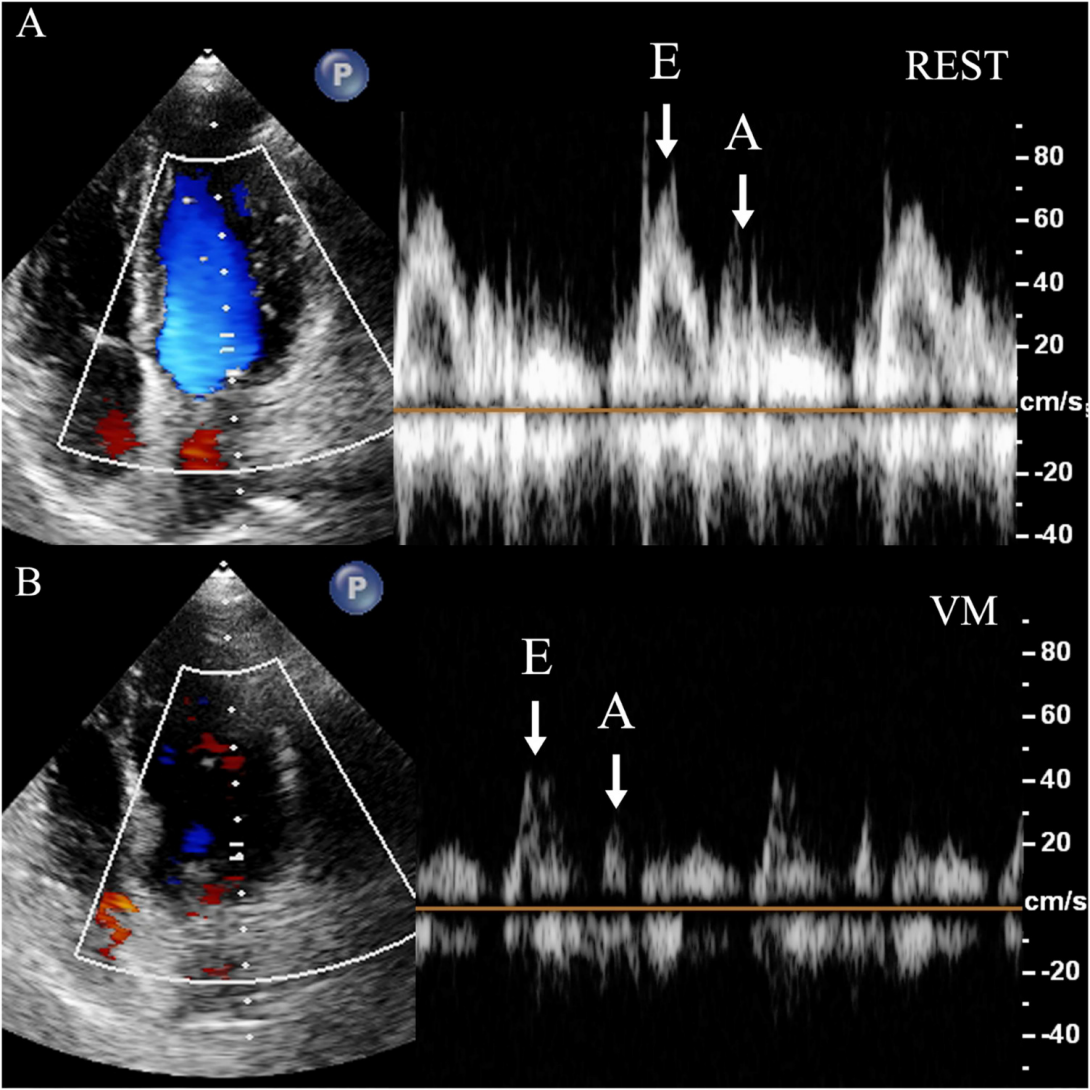
Measurement of mitral flow velocity. Dramatic decrease of the diastolic mitral flow velocity is observed. In the apical four-chamber view, the sampling volume is placed at the mitral valve orifice to measure the diastolic blood flow velocity spectrum, including the E-wave (E) and A-wave (A). **(A)** REST refers to the moment right before the subject initiates the Valsalva maneuver, when they are in a baseline or resting state. **(B)** VM refers to the point when the subject is holding their breath for 10 s during the Valsalva maneuver, just before releasing the breath. REST, at rest; VM, Valsalva maneuver; E, mitral inflow peak E-wave velocity; A, mitral inflow peak A-wave velocity.

(1) Diaphragmatic downward excursion at the roof of the right atrium (DDE-RRA): In apical four-chamber view, a sampling line for M-mode ultrasonography is positioned at the roof of the right atrium. The patient was then instructed to perform VM to measure the maximum vertical distance of the diaphragmatic downward excursion while holding their breath. This measurement is referred to as DDE-RRA (Figure 2).
(2) Diaphragmatic downward excursion at the anterior axillary line (DDE-AAL): The probe was placed at the lower edge of the lower rib in the right anterior axillary line for scanning. The patient was then instructed to perform VM to measure the maximum vertical distance of the diaphragmatic downward excursion while holding their breath. This measurement is referred to as DDE-AAL.
(3) Measurement of cardiac hemodynamic parameters: The Doppler sampling volume was placed at the tip of mitral valve or tricuspid valve. Mitral inflow peak E-wave velocity (MV-EV), mitral inflow peak A-wave velocity (MV-AV), tricuspid inflow peak E-wave (TV-EV), tricuspid inflow peak A-wave (TV-AV), mitral velocity time integral (MV-VTI), and tricuspid velocity time integral (TV-VTI) were recorded (Figure 3).
(4) Assessment of VM efficacy: Before VM execution, a disposable blowing pipeline was connected to the pressure gauge, and the oronasal forced expiration was rapidly closed when the patient performed normal inspiration. VM is considered effective when the pressure gauge indicates 40 mmHg, and this pressure must be sustained for at least 10 s.
(5) Change rate of parameters: calculated as the difference between the values at rest and during VM, divided by the value measured at rest.
(6) Grading of RLS: The number of microbubbles appearing in single-frame images of the left heart was graded as follows: Grade 0 (0 microbubbles/frame), Grade 1 (1–10 microbubbles/frame), Grade 2 (11–30 microbubbles/frame), and Grade 3 (>30 microbubbles/frame) (7).

## 3 Statistical analysis

Normally distributed continuous variables are presented as mean ± SD, whereas non-normally distributed variables are reported as medians (quartiles). Categorical variables are presented as percentages (%). Continuous variables were analyzed using the t test and Wilcoxon rank sum test and the *χ*^2^ test was used to compare the effective rate and PFO detection rate between different indicators. The Wilcoxon pair-matched test was used to compare quantitative RLS data between different measures (with Bonferroni correction). Receiver operating characteristic (ROC) curves were plotted for different indicators to determine the effectiveness of each indicator for VM. Sensitivity, specificity, and accuracy were also calculated based on the cutoff value that was maximized using the Youden index. Spearman's correlation analysis was used to describe the association between DDE-RRA and DDE-AAL. *P* values less than 0.05 (two-sided test) were considered statistically significant. All statistical analyses were performed using SPSS 26.0 software (SPSS Inc., Chicago, IL, USA).

## 4 Results

### 4.1 Basic information of the study subjects

A total of 145 patients were included in our study, including 86 males and 59 females, with an average age of 57 ± 12 years and BMI of 24 ± 6 kg/m^2^, including 52 with smoking (35.9%), 15 with diabetes (10.3%), 46 with hypertension (31.7%), 9 with coronary heart disease (6.2%), 64 with ischemic stroke (44.1%), 47 with transient ischemic attack (TIA) (32.4%), and 40 with migraine (27.6%) (Table 1).

**Table 1:** Patient characteristics.

Variables	Value
Age (y)	57 ± 12
Body mass index (kg/m^2^)	24 ± 6
Male (%)	86 (59.3)
Smoking, *n* (%)	52 (35.9)
Diabetes, *n* (%)	15 (10.3)
Hypertension, *n* (%)	46 (31.7)
Coronary heart disease, *n* (%)	9 (6.2)
Cryptogenic stroke, *n* (%)	64 (44.1)
Transient ischemic attack, *n* (%)	47 (32.4)
Migraine, *n* (%)	40 (27.6)

Data are expressed as the mean ± SD or as the number (percentage) of patients.

### 4.2 DDE-RRA comparison between AVM and non-AVM groups

**Figure 4:**
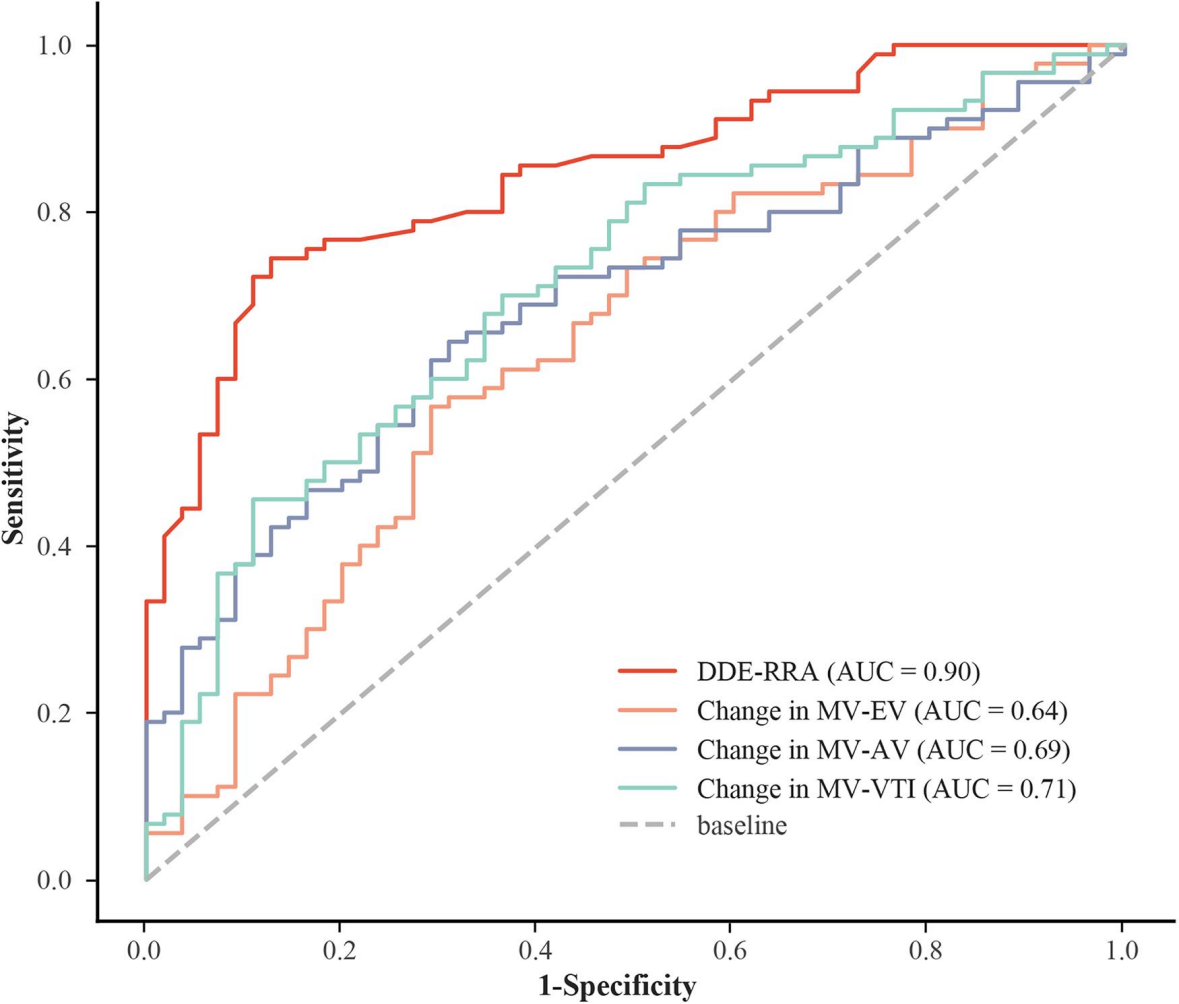
ROC for different indicators. AUC, area under the curve; DDE-RRA, diaphragmatic downward excursion at the roof of the right atrium; MV-EV, mitral inflow peak E-wave velocity; MV-AV, mitral inflow peak A-wave velocity; MV-VTI, mitral velocity time integral; ROC: receiver operating characteristic curve.

The results showed that DDE-RRA was significantly different between AVM (*n* = 90) and non-AVM group (*n* = 45) (7.3 mm vs. 3.1 mm, *P* < 0.001; Table 2). The ROC curve analysis determined that the best cutoff point for DDE-RRA is 5 mm. <5 mm was invalid for VM, while ≥ 5 mm was valid. The area under the curve (AUC) of DDE-RRA for VM effectiveness was 0.90. The sensitivity (77.8%), specificity (92.7%), positive predictive value (94.5%) and negative predictive value (70.8%) were excellent (Figure 4). Meanwhile, there was good consistency between DDE-RRA and insufflation manometry when assessing the efficacy of VM (kappa = 0.63, *P* < 0.001). The results of Spearman correlation analysis showed that there was also a significant correlation between DDE-RRA and DDE-AAL (*r* = 0.82, *P* < 0.001). These findings highlight DDE-RRA as a sensitive and clinically promising indicator for evaluating VM effectiveness.

**Table 2:** Comparison of indicators between the AVM group and the non-AVM group.

Variables	Total (*n* = 145)	non-AVM group (*n* = 55)	AVM group (*n* = 90)	*P*
Change in MV-EV (%)	17 (11, 25)	13 (9, 20)	19 (13, 27)	0.004*
Change in MV-AV (%)	17 (10, 28)	12 (8, 19)	22 (12, 31)	<0.001*
Change in MV-VTI (%)	19 (11, 27)	13 (9, 20)	21 (15, 31)	<0.001*
Change in TV-EV (%)	22 (12, 31)	21 (11, 28)	24 (14, 32)	0.142
Change in TV-AV (%)	23 (12, 33)	20 (13, 30)	25 (12, 34)	0.285
Change in TV-VTI (%)	23 (14, 34)	22 (13, 37)	24 (15, 33)	0.586
DDE-RRA (mm)	5.5 (3.2, 7.7)	3.1 (2.0, 4.4)	7.3 (5.7, 9.3)	<0.001*
DDE-AAL (mm)	26.0 (18.2, 32.9)	18.0 (13.1, 23.2)	31.7 (23.4, 35.2)	<0.001*

MV-EV, mitral inflow peak E-wave velocity; MV-AV, mitral inflow peak A-wave velocity; MV-VTI, mitral velocity time integral; TV-EV, tricuspid inflow peak E-wave velocity; TV-AV, tricuspid inflow peak A-wave velocity; TV-VTI, tricuspid velocity time integral; DDE-RRA, diaphragmatic downward excursion at the roof of the right atrium; DDE-AAL, diaphragmatic downward excursion at the anterior axillary line.

Data are expressed as the medians (quartiles). The criterion for statistical significance for all analyses was *P < 0.05.

### 4.3 Comparison of other parameters between AVM and non-AVM groups

Among all hemodynamic parameters evaluated, only MV-AV during the VM demonstrated a statistically significant difference between the AVM and non-AVM groups (*P* = 0.032; Table 3). This finding suggests that conventional parameters have limited discriminatory capacity for identifying an effective VM, highlighting the need for more reliable physiological markers. The MV-VTI had a higher specificity of 89.1%, but a lower sensitivity of 45.6%. The sensitivity and specificity of MV-AV were 64.4% and 69.1%, respectively. MV-EV had the lowest diagnostic accuracy (AUC = 0.64), low sensitivity (56.7%) and fair specificity (70.9%; Supplementary Table S1). Compared with other hemodynamic parameters, DDE-RRA showed a significantly higher diagnostic performance. Delong test confirmed that the AUC of DDE-RRA was significantly greater than those of MV-EV, MV-AV, and TV-VTI (all *P* < 0.05; Supplementary Tables S2, S3). This comparison further underscores the superior diagnostic value of DDE-RRA relative to conventional intracardiac hemodynamic parameters.

**Table 3:** AVM group and the non-AVM group difference test.

Variables	Total (*n* = 145)	non-AVM group (*n* = 55)	AVM group (*n* = 90)	*P*
MV-EV at rest (cm/s)	74.06 (63.10, 85.61)	74.06 (61.30, 84.70)	73.95 (63.26, 87.44)	0.564
MV-EV during VM (cm/s)	60.83 (52.44, 69.46)	61.08 (53.21, 75.91)	60.82 (51.04, 69.45)	0.439
MV-AV at rest (cm/s)	68.83 (57.80, 82.62)	68.83 (62.39, 76.60)	68.83 (57.70, 83.67)	0.606
MV-AV during VM (cm/s)	56.68 (47.20, 65.53)	59.82 (51.90, 66.47)	52.80 (43.21, 62.60)	0.032*
MV-VTI at rest (cm)	20.40 (16.20, 26.59)	21.04 (16.68, 26.09)	19.44 (15.83, 27.30)	0.904
MV-VTI during VM (cm)	16.10 (12.70, 22.20)	18.39 (14.30, 21.74)	15.20 (12.19, 22.50)	0.209
TV-EV at rest (cm/s)	52.41 (43.53, 63.66)	52.26 (44.61, 65.26)	53.11 (42.81, 62.16)	0.503
TV-EV during VM (cm/s)	39.62 (33.19, 50.79)	43.42 (35.74, 50.86)	38.46 (32.16, 51.02)	0.203
TV-AV at rest (cm/s)	53.95 (40.77, 64.39)	53.3 (40.12, 64.53)	54.37 (40.83, 64.36)	0.596
TV-AV during VM (cm/s)	40.93 (26.87, 51.44)	40.35 (26.77, 52.14)	41.32 (26.33, 51.18)	0.938
TV-VTI at rest (cm)	14.76 (12.32, 18.31)	14.49 (11.94, 18.6)	15.02 (12.42, 18.35)	0.589
TV-VTI during VM (cm)	11.20 (8.84, 14.64)	10.72 (8.66, 15.14)	11.28 (9.61, 14.20)	0.764

VM, Valsalva maneuver; MV-EV, mitral inflow peak E-wave velocity; MV-AV, mitral inflow peak A-wave velocity; MV-VTI, mitral velocity time integral; TV-EV, tricuspid inflow peak E-wave velocity; TV-AV, tricuspid inflow peak A-wave velocity; TV-VTI, tricuspid velocity time integral.

Data are expressed as the medians (quartiles). The criterion for statistical significance for all analyses was *P < 0.05.

### 4.4 Comparison of PFO detection rate and RLS grade

**Figure 5:**
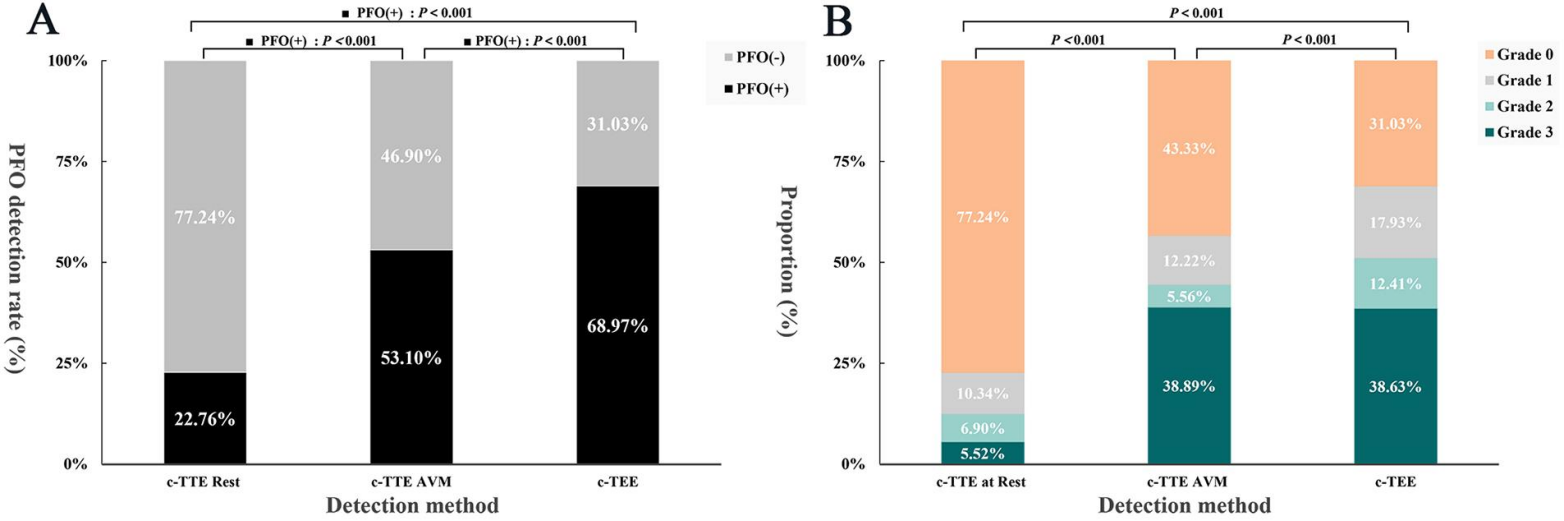
Histogram of PFO detection rate vs. RLS grade. **(A)** To compare the PFO detection rate under c-TTE at rest, c-TTE during VM, and c-TEE during VM. **(B)** To compare the RLS grade under c-TTE at rest, c-TTE during VM, and c-TEE during VM. PFO, patent foramen ovale; VM, Valsalva maneuver; c-TTE, contrast transthoracic echocardiography; c-TEE, contrast transesophageal echocardiography; RLS, right-to-left shunt.

Under c-TTE, most RLS (77.24%) were grade 0 at rest, and the proportions of grade 1, 2, and 3 RLS were 10.34%, 6.90%, and 5.52% respectively; While under VM, the proportion of grade 3 (38.89%) was significantly higher in the AVM group than at rest. And the RLS grade was also much lower in the non-AVM group than in the AVM group. There was a statistically significant difference in RLS grade between at rest, non-AVM group, and AVM group (*P* < 0.05), with more grade 2 and grade 3 RLS detected in the AVM group compared with the at rest and non-AVM group (Figure 5, Supplementary Table S4). These results demonstrate the critical influence of VM performance on RLS visualization, reinforcing the need for reliable maneuver assessment.

## 5 Discussion

This study demonstrates that objective assessment of VM performance using diaphragmatic motion is feasible in routine c-TTE examinations. The proposed DDE-RRA metric showed strong discrimination between valid and invalid maneuvers and exhibited better diagnostic performance than conventional intracardiac hemodynamic parameters. Moreover, effective VM were associated with a marked increase in detectable right-to-left shunt severity, reaffirming the necessity of reliable maneuver evaluation in PFO-related imaging. These findings highlight the potential of diaphragmatic-based assessment as a practical complementary tool for optimizing VM-dependent RLS detection.

### 5.1 Importance of provocative maneuver for accurate detection of PFO

As a non-invasive technique, c-TTE demonstrates superior compatibility with provocative maneuvers, enhancing both the detection rate of PFO and the accuracy of RLS grade assessment. This makes c-TTE a highly suitable screening method for PFO. Moreover, a suitable provocative maneuver is crucial for PFO detection. Currently, common provocative maneuvers in clinical practice are VM, intense cough, deep inspiration, abdominal compression, inferior vena cava compression and calf muscle pump tensing et al. (8–12). Spontaneous cough and hiccup can significantly elevate right atrial pressure (13), but their occurrence is accidental and difficult to cooperate with agitated saline contrast echocardiography. VM is the most effective provocative maneuver for detecting PFO (2). Kate et al. found a difference of 48.4% in the detection rate of PFO with VM compared at rest (6).VM is considered as the act of forcefully blowing through the mouth and nose for a minimum of 10 s, followed by an immediate release of the breath. Pfleger et al. compared VM with cough, deep inspiration and various expiratory pressure trials while measuring pulmonary capillary wedge pressures; the results showed that these provocative maneuvers caused an increase in pulmonary capillary wedge pressure followed by an increase in left atrial pressure, but only VM caused right atrial pressure to exceeded the pulmonary capillary wedge pressure, creating the conditions for the occurrence of PFO-RLS (14).

VM is divided into 4 stages (15). (1) During stage 1, due to increased intrathoracic pressure, pulmonary venous return is limited, and left atrial pressure can be slightly reduced; (2) during stage 2, intrathoracic pressure continues to increase, which can lead to compression of the heart and great vessels, and reduced venous return to the heart blood flow. Consequently, the right atrial preload is reduced and the intracardiac blood volume is significantly reduced, which then causes changes in intracardiac hemodynamic parameters, such as transvalvular flow velocity and VTI to varying degrees; (3) during stage 3, there is a rapid reduction in intrathoracic pressure with the release of breath. This causes an immediate increase in blood returning to the heart and the right atrial pressure to increase. At the same time, the pressure in the left atrium immediately decreased, while the pressures in the left and right ventricles were reversed. This caused the right atrial pressure to exceed the left atrial pressure. If PFO was present, RLS occurred during this period. Eventually, this pressure reversal gradually returned to normal during stage 4. (4) During stage 4, cardiac hemodynamics gradually returned to normal respiratory status.

The assessment of VM in clinical practice relies mostly on visual and numerical evaluations under TTE (16). In terms of visual evaluation, we used visual observation of the displacement of the interatrial septum to the left atrial side during VM release phase to determine the effectiveness of VM. Because of the individual differences in the size and extent of the fossa ovalis, interatrial septum with a large range of motion is more likely to be observed bulging to the left atrial side during VM, but the sensitivity of this method is poor, and the swing amplitude of most interatrial septum under TTE is not sufficient to determine the efficacy of VM, resulting in biased assessment results. In numerical quantitative evaluation, the efficacy of VM can be assessed by measuring changes in MV-EV and MV-AV, or by insufflation manometry, but these methods have large individual differences or require external devices that are not convenient enough. Measurement of DDE provides a new perspective to quantitatively assess the efficacy of VM.

Invasive right atrial pressure measurement remains the strict physiological gold standard for evaluating VM. Nevertheless, since Martin Flack first introduced the 40 mmHg intrathoracic pressure threshold in 1919 using a mercury sphygmomanometer (17), insufflation manometry at this level has been extensively validated and widely adopted as a pragmatic clinical reference standard in routine practice. DDE provides a novel, objective perspective for assessing VM efficacy; however, its application remains technically challenging and subject to individual variability. Therefore, DDE should currently be regarded as a complementary tool for the diagnostic evaluation of right-to-left shunting in PFO. In our study, the selected patient cohort did not undergo invasive catheterization, which may limit the reliability of our findings. This work therefore represents a preliminary attempt, and future investigations, particularly in PFO closure patients undergoing right heart catheterization, are warranted to further validate the DDE-RRA method.

### 5.2 The role of DDE in the assessment of the efficacy of VM

The diaphragm is the main respiratory muscle of the human body, located between the thoracic and abdominal cavities, adjacent to the pleural cavity, lung, and pericardial cavity. Its displacement is affected by age, body position, and respiratory pattern (18, 19). Diaphragmatic excursion has a linear relationship with inspiratory capacity; therefore, diaphragmatic function can be assessed quantitatively and qualitatively by measuring the changes in diaphragmatic excursion amplitude (20). Ultrasound is recognized as a powerful tool for the assessment of diaphragmatic function, with the advantages of being non-invasive, rapid and reproducible (21). When diaphragmatic dysfunction is suspected, ultrasound is recommended as the preferred technique for assessing diaphragmatic motion. Currently, the diaphragmatic roof view through the anterior axillary line is used to assess the functional status of the diaphragm, and changes in the diaphragmatic excursion amplitude and time can be assessed using M-mode ultrasonography (22). However, it was not possible to perform TTE at the same time as this view, so our study attempted to measure DDE in apical four-chamber view under TTE. We found that the DDE moved significantly excursions with VM and was not easily off-target. DDE-RRA was measured in this view, which not only allows simultaneous observation of RLS, but also does not require repositioning of the patient. At the same time, M-mode ultrasound has a good temporal resolution and can record the DDE at the moment of VM release in time (23). Therefore, our study innovatively proposed the use of M-mode ultrasound to measure DDE as an evaluation indicator for efficacy of VM. In our study, the correlation analysis of DDE-RRA and DDE-AAL was also performed and the results showed that there was a positive correlation between the two and the correlation was good (*r* = 0.843, *P* < 0.05), therefore it was considered that the assessment of diaphragmatic excursion could be performed by measuring DDE-RRA instead of DDE-AAL.

DDE during VM is a complex process. During stage 1–2 (forced expiration), intrapleural pressure is increased by the action of the expiratory muscles (abdominal and internal intercostal muscles). Research has demonstrated that when the glottis is shut and forced to expel air, the intrapleural pressure receives quick adjustment and increases from its initial negative pressure to 110 mmHg, causing the DDE. Despite the increase in abdominal pressure, which exerts an upward force on the diaphragm, the intrapleural pressure on the diaphragm was significantly higher than the abdominal pressure. Additionally, contraction of the diaphragm causes downward excursion due to a downward force (24). During stage 3, intrapleural pressure decreases instantaneously in the release. At this instant, the diaphragm was reversed and raised.

The results of our study showed that there was a significant difference in DDE-RRA between the AVM and non-AVM groups (7.3 mm vs. 3.1 mm, *P* < 0.001). The DDE-RRA cutoff value of the ROC curve was calculated to be 5 mm, while the AUC of DDE-RRA for assessing the efficacy of VM was 0.90, and the sensitivity and specificity were 77.8% and 92.7%, respectively. At the same time, there was a good consistency between DDE-RRA and insufflation manometry in judging the efficacy of VM (kappa = 0.63, *P* < 0.001). Therefore, our study has shown that DDE-RRA has a high diagnostic efficacy in assessing the efficacy of VM, which in turn facilitates the effective detection of PFO. In our study, two methods were used to measure DDE-RRA. The first method involved measuring the difference between the diaphragm position at the end of VM stage 2 and its position at rest. The second method involved measuring the difference between the diaphragm position at the end of VM stage 2 and its position after VM was released and returned to rest. Nevertheless, because of the patient's significant variation in breathing intensity after breath hold release, it requires a considerable amount of time for them to regain a condition at rest, which makes the second method difficult and inefficient for measurement. Therefore, the first method was chosen to measure DDE-RRA in our study. Moreover, it should be noted that inter-patient anatomical variability, such as differences in diaphragmatic development, obesity, or thoracic compliance, may influence DDE measurements. These anatomical and physiological differences could partly explain individual variability in DDE responses to VM. Although our study minimized confounding by excluding patients with poor acoustic windows, future studies may consider more detailed subgroup analyses to further clarify the influence of these factors.

In clinical practice, VM can be used to enhance the detection rate of PFO. Additionally, VM can also be employed to assess various clinical examinations that involve quantifying the magnitude of respiratory motion. Examples of such examinations include measuring the flow velocity gradient during hypertrophic cardiomyopathy excitation, conducting pelvic floor ultrasonography, and evaluating saphenofemoral valve function (25–27). The DDE proposed in our study is expected to assist in the evaluation of these examinations, especially the evaluation of occult obstruction in hypertrophic cardiomyopathy.

### 5.3 Comparison of DDE-RRA with other parameters of assessing the efficacy of VM

Variations in intrathoracic pressure are responsible for variations in intravascular hemodynamics during VM. Intrapleural pressure, intrapulmonary pressure, and muscle activity all influence variations in intrathoracic pressure, subsequently, resulting in alterations in intracardiac parameters. During VM, both intrapleural pressure and intrapulmonary pressure increased. This leads to an increase in intrathoracic pressure, causing DDE and compress the heart and great vessels located in the thoracic cavity. The increased intrathoracic pressure also resulted in a blockage of venous return, leading to a decrease in MV-EV, MV-AV, TV-EV, TV-AV, MV-VTI, TV-VTI. Enfa Zhao et al. found that the MV-EV reduced by 18.0%, MV-AV decreased by 10.6%, and there were no significant changes in TV-EV, TV-AV, TV-VTI, and E/A ratio when adequate VM was done (28). We found that only MV-AV during the VM showed a statistically significant difference between the AVM and non-AVM groups, indicating that conventional parameters provide limited specificity and are not reliable indicators for assessing VM effectiveness.

Compared to blood flow spectrum measurement, DDE-RRA measurement is more convenient and efficient. And the diaphragm at the roof of the atrium showed well in apical four-chamber view and maintained high detection accuracy even when the quality of the cardiac ultrasound image was poor. Therefore, DDE-RRA is the convenience and reliable indicator for the evaluation of the efficacy of VM under TTE.

### 5.4 Limitations

This study has several limitations. First, the cohort primarily included patients referred with a high suspicion of PFO, which may limit the applicability of our findings to lower-prevalence populations or general screening contexts. Future studies should examine the diagnostic performance of DDE-RRA in broader, less selected populations. Second, as this work represents an initial validation of the DDE-RRA method, it was conducted at a single center to maintain consistency in imaging protocols and operator techniques. Although this minimized methodological variability, it may also restrict external validity. Multicenter studies with larger and more diverse cohorts are warranted. Third, the absence of invasive right atrial pressure validation constitutes an important limitation, as this remains the physiological gold standard for evaluating VM efficacy. Future investigations incorporating invasive assessment are needed to confirm the reliability of our findings. Finally, we assessed DDE exclusively during the VM and did not evaluate its performance under other provocative maneuvers such as coughing, abdominal compression, or inferior vena cava compression.

## 6 Conclusions

This study introduces DDE as a novel and practical tool for assessing VM efficacy, offering simplicity, real-time feasibility, and potential to complement conventional PFO assessment. This work should be regarded as a preliminary attempt, highlighting the promise of this approach. Future studies incorporating invasive right atrial pressure validation will be essential to confirm and expand its clinical applicability.

## Data Availability

The original contributions presented in the study are included in the article/Supplementary Material, further inquiries can be directed to the corresponding author/s.
