# Supplementary material for: Diaphragmatic downward excursion as a novel metric for assessing Valsalva maneuver efficacy in patent foramen ovale detection by contrast transthoracic echocardiography
Source: Front Cardiovasc Med. 2025 Dec 19;12:1616241. doi: 10.3389/fcvm.2025.1616241 (PMC12771766; doi:10.3389/fcvm.2025.1616241)
Supplement: Supplementary file 1 [file Datasheet1.docx]

**SUPPLEMENTARY DATA: Frontiers in Cardiovascular Medicine**

**A DIFFERENCE THAT MATTERS: THE AORTIC ROOT ANATOMY OF LARGE ANIMAL MODELS VERSUS HUMANS**

Venessa Strauss^1^, Harish Appa^1^, Paul Human^2,3^, Ferdinand Vogt^4,5^, Waled Hadasha^1^, Jacques Scherman^3^, Qonita Said-Hartley^6^, Yvonne Schneeberger^7^, Helga Bergmeister^8,9^, Lenard Conradi^10^, Bruno Podesser^8,9,11^, Peter Zilla^1,2,3^

**MATERIALS AND METHODS:**

***Ex-Vivo Silicone Casting***

Aortic roots of Merino and Dorper sheep, as well as Landrace pigs and Kolbroek mini-pigs, were sourced from abattoirs registered with the Department of Agriculture, Land Reform and Rural Development of South Africa. Approval from the local animal ethics committee was not required, as hearts were collected following slaughter inherent to commercial meat production for human consumption, independent of study requirements.

Aortic roots from Yorkshire pigs and Aachen mini pigs were obtained from experimental animals of the Center for Biomedical Research and Translational Surgery, Medical University Vienna, that had reached the termination point of unrelated approved research projects (Team and individual training for surgical procedures in a large animal model, Protocol No. GZ 66.009/0068-V/3b/2019, Evaluation of minipigs as a large animal model in hearing research and cochlear implantation, Protocol No. GZ 2020-0.272.252) (Table I). No animals were sacrificed specifically for heart procurement to produce casts.

Experimental animals were euthanised under deep general anaesthesia induced by intramuscular injection of Medetomidine (0.1 mg/kg) and Ketamine (10 mg/kg), followed by Propofol (1 mg/kg), and maintained with inhaled Sevoflurane (2–4%) with a rapid intravenous bolus of Potassium Chloride (150 mg/kg) was administered to ensure humane termination.

***In vivo*: ECG-gated Computed Tomography (CT) (Table II)**

CT scans were obtained from animals involved in unrelated research projects approved by the Animal Ethics Committees of the Faculty of Health Sciences at the UCT (Acute Pig Trial in Preparation for Human Clinical Testing Protocol No. 020-015, and Evaluation of Thrombogenicity and Haemodynamic Performance of a Transcatheter Polymer-leaflet Aortic Valve Replacement (TAVR) System in Döhne Merino Sheep Protocol No. 023-032), and the University of Vienna (MA: 2023-0.798.268: Long-Term Evaluation of a Polymeric Prosthetic Aortic Valve in a Chronic Pig Model Protocol No. GZ 66.009/0068-V/3b/2019).

The study of healthy patient data was approved by the Faculty of Health Sciences Surgical Department Research Committee, University of Cape Town (In vitro and In silico Evaluation of Three-Dimensional Computed Tomography Geometry of the Aortic Root of Patients in the Archives of the Dept. of Radiology Project No. 2024/243). The study of AS patient data was approved by the Health Research Ethics Committee, University of Cape Town (In-vitro and In-silico Evaluation of Three-Dimensional Computed Tomography Geometry of the Aortic Root in Patients Screened for Transcatheter Aortic Valve Implantation Reference No. 826/2020). Ethics approval from the University Hospital Hamburg was not required, as deidentified images were supplied. (17)

**RESULTS**

|  | **SHEEP** | | | **PIGS** | | | | **MINI PIGS** | |
| --- | --- | --- | --- | --- | --- | --- | --- | --- | --- |
|  | **Merino** | | **Dorper** | **Landrace** | | **Yorkshire** | | **Kolbroek** | **Aachen** |
|  | **Abattoir** | **Termination** | **Abattoir** | | **Abattoir** | | **Termination** | **Termination** | **Termination** |
| N | 10 | 10 | 10 | | 10 | | 10 | 10 | 6 |
| Age Bracket (mo) | ±9 | ±12 | ±3-4 | | ±4-6 | | 3-4 | 9-12 | 9-12 |
| **Weight (kg)** | 35-40 | 35-45 | 30-40 | | 65-80 | | 35-45 | 40-65 | 35-40 |
| Mean | 36.3 | 40.8 | 34.7 | | 74.8 | | 40.8 | 50.9 | 38.8 |
| StdDev | 2.1 | 6.7 | 2.8 | | 4.8 | | 5.9 | 10.6 | 1.6 |
| *Median | 36.6 | 40.0 | 33.8 | | 75.4 | | 42.5 | 51.0 | 39.5 |
| *Range | 33.4-39.4 | 30.0-52.0 | 32.3-41.9 | | 66.0-82.5 | | 32.0-50.0 | 38.0-66.0 | 36.0-40.0 |
| **Annulus Diameter (mm)** |  |  |  | |  | |  |  |  |
| Mean | 22.0 | 22.1 | 20.6 | | 26.5 | | 20.2 | 22.8 | 18.0 |
| StdDev | 1.7 | 2.3 | 1.4 | | 2.2 | | 2.1 | 2.0 | 0.9 |
| *Median | 21.7 | 21.4 | 20.3 | | 28.0 | | 20.6 | 22.4 | 17.8 |
| *Range | 19.9-24.4 | 19.4-26.5 | 18.6-22.7 | | 23.6-28.5 | | 16.9-23.4 | 20.2-26.0 | 17.1-19.3 |
| **Hegar Diameter (mm)** |  |  |  | |  | |  |  |  |
| Mean | 23.4 | 24.7 | 23.5 | | 27.6 | | 22.9 | 23.4 | 21.8 |
| StdDev | 1.0 | 1.6 | 1.7 | | 1.8 | | 1.7 | 1.8 | 1.0 |
| *Median | 23.5 | 24.0 | 23.0 | | 27.0 | | 23.0 | 23.0 | 22.0 |
| *Range | 22.0-25.0 | 22.0-27.0 | 22.0-28.0 | | 26.0-30.0 | | 21.0-26.0 | 22.0-27.0 | 20.0-23.0 |
| **SOV Diameter – 3D (mm)** |  |  |  | |  | |  |  |  |
| *Mean | 26.0 | 26.3 | 24.5 | | 31.3 | | 23.1 | 26.4 | 20.1 |
| *StdDev | 2.9 | 2.5 | 2.0 | | 3.6 | | 2.8 | 2.9 | 0.7 |
| Median | 26.3 | 26.9 | 24.6 | | 31.3 | | 22.9 | 26.5 | 20.1 |
| Range | 21.7-29.7 | 21.0-29.3 | 20.0-27.4 | | 27.2-38.4 | | 18.5-28.4 | 21.1-30.0 | 19.1-21.1 |
| **SOV Diameter - Clin (mm)** |  |  |  | |  | |  |  |  |
| Mean | 22.1 | 22.2 | 20.9 | | 26.5 | | 19.9 | 22.8 | 17.7 |
| StdDev | 2.2 | 2.0 | 1.5 | | 2.7 | | 2.5 | 2.3 | 0.6 |
| Median | 22.3 | 22.7 | 21.2 | | 26.7 | | 19.7 | 23.0 | 17.7 |
| Range | 19.9-25.0 | 18.1-24.6 | 17.7-22.4 | | 23.3-31.4 | | 16.9-25.6 | 18.6-26.1 | 17.0-18.4 |
|  |  |  |  | |  | |  |  |  |
| **STJ Diameter (mm)** |  |  |  | |  | |  |  |  |
| *Mean | 19.0 | 20.1 | 17.5 | | 24.0 | | 18.0 | 17.0 | 15.1 |
| *StdDev | 2.4 | 2.2 | 2.0 | | 2.5 | | 1.7 | 2.0 | 1.0 |
| Median | 18.8 | 20.4 | 18.3 | | 23.5 | | 18.2 | 17.3 | 15.1 |
| Range | 15.8-22.8 | 15.9-23.4 | 13.7-19.5 | | 20.3-28.7 | | 15.4-20.9 | 13.9-20.2 | 14.0-16.7 |
| **Ann-STJ Height (mm)** |  |  |  | |  | |  |  |  |
| Mean | 13.2 | 12.8 | 13.9 | | 18.6 | | 14.1 | 17.3 | 12.4 |
| StdDev | 1.9 | 1.1 | 0.8 | | 2.4 | | 1.2 | 3.2 | 1.5 |
| *Median | 13.7 | 12.9 | 13.7 | | 18.8 | | 14.1 | 17.2 | 12.9 |
| *Range | 10.2-15.8 | 11.2-14.5 | 12.8-15.5 | | 15.2-22.2 | | 11.9-15.7 | 11.4-21.2 | 10.5-14.1 |
| **Ann:SOV Ratio** |  |  |  | |  | |  |  |  |
| *Mean | 0.85 | 0.85 | 0.84 | | 0.85 | | 0.88 | 0.87 | 0.90 |
| *StdDev | 0.08 | 0.07 | 0.05 | | 0.06 | | 0.04 | 0.09 | 0.05 |
| Median | 0.82 | 0.83 | 0.84 | | 0.85 | | 0.89 | 0.89 | 0.89 |
| Range | 0.78-0.99 | 0.74-0.96 | 0.75-0.93 | | 0.74-0.99 | | 0.81-0.93 | 0.75-0.99 | 0.84-0.97 |
| **LC) Height (Lower) (mm)** |  |  |  | |  | |  |  |  |
| Mean | 3.0 | 3.6 | 2.7 | | 4.0 | | 4.3 | 5.8 | 1.9 |
| StdDev | 1.0 | 0.7 | 0.9 | | 1.3 | | 2.2 | 1.8 | 0.8 |
| *Median | 2.7 | 3.6 | 2.7 | | 4.0 | | 4.1 | 5.9 | 1.7 |
| *Range | 1.8-4.6 | 2.2-4.4 | 1.5-4.1 | | 2.4-5.9 | | 1.8-8.0 | 2.3-8.0 | 1.3-3.5 |
| **LCO Diameter (mm)** |  |  |  | |  | |  |  |  |
| *Mean | 7.4 | 7.0 | 7.2 | | 8.9 | | 5.8 | 6.0 | 7.0 |
| *StdDev | 1.2 | 1.0 | 0.9 | | 1.3 | | 2.2 | 1.5 | 1.2 |
| Median | 7.7 | 6.9 | 7.3 | | 8.7 | | 5.8 | 5.5 | 6.8 |
| Range | 5.3-9.2 | 5.7-8.8 | 5.7-8.3 | | 7.2-11.5 | | 1.7-9.4 | 4.6-9.2 | 5.7-8.4 |
| **LCO Area (mm^2^)** |  |  |  | |  | |  |  |  |
| *Mean | 30.8 | 31.5 | 33.8 | | 37.1 | | 31.4 | 22.5 | 32.0 |
| *StdDev | 8.4 | 8.1 | 8.0 | | 7.5 | | 10.4 | 3.8 | 6.1 |
| Median | 29.0 | 32.2 | 33.1 | | 40.4 | | 32.0 | 21.4 | 30.7 |
| Range | 19.5-47.6 | 21.1-46.2 | 20.5-50.7 | | 26.2-45.4 | | 16.9-44.8 | 17.9-28.3 | 24.3-39.4 |
| **Eccentricity LCO (˚)** |  |  |  | |  | |  |  |  |
| Mean | 26.4 | 26.0 | 26.2 | | 29.1 | | 18.2 | 27.0 | 21.9 |
| StdDev | 6.8 | 4.2 | 7.7 | | 4.6 | | 6.8 | 7.7 | 5.1 |
| *Median | 26.2 | 25.3 | 26.8 | | 29.3 | | 18.1 | 26.6 | 20.9 |
| *Range | 17.2-37.8 | 21.5-35.4 | 11.3-37.4 | | 21.2-34.7 | | 8.6-26.7 | 12.3-42.7 | 16.7-30.9 |
| **RCO Height (mm)** |  |  |  | |  | |  |  |  |
| Mean | 6.2 | 6.4 | 6.7 | | 10.0 | | 7.6 | 11.3 | 7.1 |
| StdDev | 1.4 | 1.3 | 1.5 | | 3.1 | | 1.5 | 2.9 | 1.6 |
| *Median | 6.5 | 6.4 | 6.4 | | 8.5 | | 8.1 | 11.7 | 7.1 |
| *Range | 3.9-7.9 | 4.5-9.3 | 5.0-9.0 | | 6.9-15.6 | | 4.9-9.4 | 6.9-16.2 | 5.2-8.9 |
| **RCO Diameter (mm)** |  |  |  | |  | |  |  |  |
| Mean | 3.8 | 3.9 | 4.0 | | 6.6 | | 4.9 | 4.3 | 3.4 |
| StdDev | 1.3 | 0.9 | 0.9 | | 1.7 | | 1.7 | 1.1 | 0.7 |
| *Median | 3.7 | 3.8 | 4.2 | | 6.0 | | 4.2 | 4.5 | 3.4 |
| *Range | 1.9-5.8 | 2.6-5.3 | 2.8-5.6 | | 4.4-9.7 | | 3.7-9.4 | 2.4-5.9 | 2.6-4.6 |
| **RCO Area (mm^2^)** |  |  |  | |  | |  |  |  |
| Mean | 3.8 | 3.9 | 4.0 | | 6.6 | | 4.9 | 4.3 | 3.4 |
| StdDev | 1.3 | 0.9 | 0.9 | | 1.7 | | 1.7 | 1.1 | 0.7 |
| *Median | 3.7 | 3.8 | 4.2 | | 6.0 | | 4.2 | 4.5 | 3.4 |
| *Range | 1.9-5.8 | 2.6-5.3 | 2.8-5.6 | | 4.4-9.7 | | 3.7-9.4 | 2.4-5.9 | 2.6-4.6 |
| **Eccentricity RCO (˚)** |  |  |  | |  | |  |  |  |
| Mean | 6.2 | 0.8 | 0.9 | | -2.1 | | -5.0 | -5.2 | -2.3 |
| StdDev | 8.0 | 8.3 | 8.6 | | 3.5 | | 8.6 | 12.6 | 6.6 |
| *Median | 3.3 | 1.3 | -1.6 | | -1.5 | | -2.9 | -2.2 | -1.4 |
| *Range | -6.5:8.0 | -13.6:15.1 | -8.7:18.7 | | -9.2:3.9 | | -19.6:4.4 | -33.6:9.2 | -10.2:5.1 |

***Table III:*** *Dimensional analysis of silicone casts of aortic roots comparing different species and breeds, as well as the method of tissue harvest—either from abattoir-sourced specimens ("Abattoir") or on-table collection at the termination of experimental procedures ("Termination").*

|  | **HUMANS** | | | | | | | **PIGS** | | | | | | **SHEEP** | | | | | | |
| --- | --- | --- | --- | --- | --- | --- | --- | --- | --- | --- | --- | --- | --- | --- | --- | --- | --- | --- | --- | --- |
|  | **Healthy** | | **AR** | | **AS** | | | **Landrace** | | | **Yorkshire** | | | **Merino** | | **Merino** | | **Merino** | |  |
|  | **3D** | **Clin** | **3D** | **Clin** | **3D** | **Clin** | **3D** | | **Clin** | **3D** | | **Clin** | **3D** | | **Clin** | **3D** | **Clin** | **3D** | **Clin** |  |
| N | 10 | | 10 | | 9 | | | 5 | | | 8 | | | 8 | | 8 | | 3 | |  |
| Age | 43±5 yr | | 74±11 yr | | 79±5 yr | | | 3-4 mo | | | 3-4 mo | | | 9 mo | | 12 mo | | 12-18 mo | |  |
| Weight (kg) | 78.7±17.9 | | 71.0±17.0 | | 65.9±10.8 | | | 61.0±5.5 | | | 46.5±5.8 | | | 23.9±1.9 | | 40.4±2.1 | | 81.3±5.5 | |  |
| Annulus Diameter (mm) | 25.8 ±2.2 | 25.7 ±1.9 | 27.7 ±2.8 | 26.9 ±2.4 | 23.3 ±2.4 | 24.0 ±1.8 | 23.9 ±1.5 | | 24.6 ±1.8 | 24.7 ±1.8 | | 24.9 ±3.0 | 25.1 ±1.1 | | 24.5 ±1.1 | 26.3 ±1.2 | 26.8 ±1.4 | 32.6 ±3.1 | 31.5 ±1.3 |  |
| SOV Diameter (mm) | 35.3 ±3.7 | 31.4 ±2.9 | 39.4 ±5.7 | 34.6 ±5.1 | 30.9 ±2.8 | 27.3 ±2.7 | 32.6 ±3.2 | | 28.2 ±1.9 | 34.6 ±3.7 | | 28.9 ±3.0 | 31.5 ±1.4 | | 27.5 ±1.3 | 33.6 ±4.0 | 29.2 ±3.1 | 37.6 ±3.2 | 33.3 ±2.2 |  |
| Ann:SOV (ratio) | 0.7 ±0.1 | 0.8 ±0.01 | 0.7 ±0.01 | 0.8 ±0.1 | 0.7 ±0.1 | 0.9 ±0.01 | 0.7 ±0.0 | | 0.9 ±0.03 | 0.7 ±0.1 | | 0.9 ±0.1 | 0.8 ±0.1 | | 0.9 ±0.1 | 0.8 ±0.1 | 0.9 ±0.1 | 0.9 ±0.0 | 0.9 ±0.0 |  |
| Ann:STJ (ratio) | 0.91 ±0.06 | 0.91 ±0.05 | 0.88 ±0.12 | 0.85 ±0.11 | 0.91 ±0.06 | 0.94 ±0.06 | 0.99 ±0.10 | | 1.00 ±0.08 | 0.99 ±0.11 | | 1.00 ±0.10 | 0.99 ±0.07 | | 0.97 ±0.05 | 1.05 ±0.11 | 1.07 ±0.10 | 1.05 ±0.03 | 1.04 ±0.06 |  |
| STJ Diameter (mm) | 28.4±2.2 | | 32.0±4.5 | | 25.7±2.2 | | | 24.5±2.0 | | | 25.0±2.3 | | | 25.4±1.2 | | 24.5±2.7 | | 30.8±2.0 | |  |
| STJ Height (mm) | 21.3±2.4 | | 22.6±6.4 | | 20.7±2.6 | | | 21.3±4.5 | | | 19.7±2.4 | | | 16.5±3.3 | | 17.2±2.3 | | 20.1±2.8 | |  |
| LCO Height (Lower) (mm) | 10.9±3.0 | | 10.3±4.4 | | 9.7±2.6 | | | 3.2±2.6 | | | 3.0±3.0 | | | 2.0±0.9 | | 3.1±1.6 | | 3.9±0.7 | |  |
| LCO Height (Ave Top-Bottom) (mm) | 14.8±3.1 | | 14.5±3.9 | | 13.0±2.5 | | | 8.4±3.7 | | | 9.1±3.0 | | | 7.7±1.3 | | 8.2±1.3 | | 9.2±1.0 | |  |
| LCO Diameter (mm) | 7.7±2.7 | | 8.4±1.6 | | 6.7±1.3 | | | 11.5±2.2 | | | 12.1±3.1 | | | 11.5±1.6 | | 10.2±2.5 | | 10.7±2.4 | |  |
| LCO Area (mm^2^) | 51.7±37.6 | | 57.3±22.3 | | 36.6±13.6 | | | 107.0±43.5 | | | 122.2±59.9 | | | 104.7±29.0 | | 85.6±42.9 | | 92.7±38.0 | |  |
| LCO Eccentricity (°) | 8.2±5.5 | | 7.3±3.5 | | 9.9±6.8 | | | 17.5±6.7 | | | 19.1±6.2 | | | 17.7±9.5 | | 24.0±4.6 | | 22.0±10.2 | |  |
| RCO Height (Lower mm) | 13.2±2.4 | | 12.6±5.2 | | 13.2±1.8 | | | 10.8±4.8 | | | 10.9±2.1 | | | 9.3 =±4.0 | | 9.5±2.9 | | 13. ±2.2 | |  |
| RCO Height (Ave top-bottom) (mm) | 15.9±2.3 | | 16.0 ±5.2 | | 15.8 ±2.0 | | | 14.6 ±4.5 | | | 14.1 ±2.1 | | | 11.5 ±3.8 | | 12.2 ±2.5 | | 15.8 ±2.4 | |  |
| RCO Diameter (mm) | 5.3±1.6 | | 6.7 ±1.4 | | 5.2 ±0.9 | | | 7.6 ±1.9 | | | 6.4 ±0.6 | | | 4.5 ±1.5 | | 5.3 ±2.1 | | 5.1 ±0.7 | |  |
| RCO Area (mm^2^) | 14.2±7.1 | | 15.4±8.6 | | 13.9±6.2 | | | 27.7±17.3 | | | 29.4±10.7 | | | 10.2±6.6 | | 18.9±8.2 | | 17.2±8.7 | |  |
| RCO Eccentricity (°) | 13.2±9.7 | | 12.4 ±10.1 | | 14.7 ±12.2 | | | 11.5 ±5.6 | | | 10.7 ±7.0 | | | 12.2 ±10.5 | | 9.8 ±7.3 | | 5.9 ±1.8 | |  |

***Table IV:*** *CT-based comparison of key aortic root dimensions using the conventional 2D CT-based clinical measurement method versus 3D reconstruction-based assessment. As most data were skewed and analysed using non-parametric tests, values are presented as median ± range. Mean ± standard deviation (SD) is shown in italics for reference.*

**Abbreviations:**

AR (aortic regurgitation), AS (aortic stenosis), RCO (right coronary ostia), LCO (left coronary ostia), SOV (sinuses of Valsalva), STJ (sinotubular junction).
